# Predominance of In‐Field Recurrence After Radiotherapy for Sinonasal Cancer: A Single‐Center Retrospective Study

**DOI:** 10.1002/hed.70287

**Published:** 2026-04-23

**Authors:** M. de Ridder, L. J. van de Velde, T. de Bie, R. van Noortwijk, C. P. J. Raaijmakers, E. H. Voormolen, W. W. Braunius, R. de Bree, G. E. Breimer, L. A. Devriese, E. J. Smid, J. A. Rijken

**Affiliations:** ^1^ Department of Radiation Oncology University Medical Center Utrecht Utrecht the Netherlands; ^2^ Department of Head and Neck Surgical Oncology University Medical Center Utrecht Utrecht the Netherlands; ^3^ Department of Neurosurgery University Medical Center Utrecht Utrecht the Netherlands; ^4^ Department of Pathology University Medical Center Utrecht Utrecht the Netherlands; ^5^ Department of Medical Oncology University Medical Center Utrecht Utrecht the Netherlands

**Keywords:** malignancy, radiotherapy, recurrence, sinonasal cancer

## Abstract

**Background:**

Sinonasal malignancies (SNM) are rare, heterogeneous tumors with poor prognosis. There is an unmet need to improve treatment outcomes. Despite advances in imaging and molecular classification, optimal curative radiotherapy (RT) strategies remain undefined. We analyzed the pattern of failure after RT, focusing in depth on local recurrence.

**Methods:**

This retrospective cohort study included 104 SNM patients treated with curative‐intent RT (either primary or after endoscopic surgery) at a tertiary center between 2010 and 2022. Local recurrences were delineated on follow‐up imaging and registered to pretreatment plans. Recurrence location was classified as in‐field, marginal, or out‐of‐field based on dose at the center of mass.

**Results:**

Local recurrence rate was 25%. Most local recurrences (81%) occurred in‐field in the high‐dose region (> 95% of prescribed dose), with a median dose of 65 Gy. Perineural spread accounted for most out‐of‐field failures. Local recurrence significantly impaired disease‐specific survival.

**Conclusions:**

Local failures after RT for SNM predominantly arise in‐field within adequately irradiated volumes, suggesting biological radioresistance. These findings support the need for histology‐driven, biologically tailored treatment strategies.

## 1 Introduction

Sinonasal malignancies (SNM) are rare tumors, with an estimated annual incidence of 130–150 cases in the Netherlands [1]. They are associated with a poor prognosis, partly because of often initial nonspecific symptoms mimicking benign conditions such as sinusitis, leading to diagnostic delays of approximately 6–8 months [2, 3, 4, 5, 6]. Consequently, patients often present with advanced‐stage disease. The heterogeneity of SNM further complicates treatment, as the sinonasal cavities encompass a complex histological landscape. The mucosal lining contains diverse epithelial cell types, including sustentacular cells, basal cells, and olfactory neurons [7, 8]. Beneath the mucosa, the lamina propria harbors glands, immune cells, and an extensive vascular network. This rich environment predisposes to the variation of malignancies found in sinonasal tumors.

Risk factors vary according to histological subtype. For instance, exposure to wood and leather dust has been linked to intestinal‐type adenocarcinoma, whereas human papillomavirus (HPV) and smoking are associated with squamous cell carcinoma (SCC) [9, 10]. For several subtypes, however, no clear etiological factors have been identified.

Recent advances in molecular biology and genetics, such as mutation and methylation profiling, are guiding current therapeutic choices. These developments are reflected in the updated WHO classification of nasal, paranasal, and skull base tumors, emphasizing the role of gene aberrations, such as *IDH1/2*, *SWI/SNF*, *NUTM1*, and *DEK*::*AFF2*, alongside histopathological features [11, 12, 13].

Survival outcomes depend heavily on tumor histology, underscoring the need for tailored treatment strategies [11, 13]. Histology‐driven approaches have already altered patient management—for instance, in sinonasal mucosal melanoma (SNMM), which exhibits high recurrence rates necessitating rigorous follow‐up [14, 15, 16]. Treatment is increasingly histology‐driven, although the standard of care for most SNM still consists of surgery with adjuvant therapy. In the majority of SNM, adjuvant treatment contains radiotherapy (RT). For unresectable SNM, RT is the backbone of treatment with induction and/or concurrent chemotherapy.

A key issue in RT for SNM is the lack of consensus on target volume delineation, dose, and fractionation schedules. One of the reasons for that is that prospective clinical trials remain challenging due to the low incidence and heterogeneity of SNM. This leaves key RT questions, such as the spatial patterns of local failure, insufficiently characterized in contemporary practice. Consequently, retrospective analyses with detailed patient data are vital for informing clinical practice.

In this study, we aimed to analyze failure patterns following curative (either primary or postoperative) RT in SNM patients, with particular focus on local recurrences, to inform future treatment strategies.

## 2 Patients and Methods

### 2.1 Study Population

This single‐center retrospective cohort study included 104 consecutive patients with primary SNM treated with curative‐intent RT (either primary or postoperative) at the University Medical Center Utrecht (UMCU), a tertiary referral center for head and neck cancer. Patients were diagnosed between January 2010 and April 2022, allowing at least 24 months of follow‐up. Follow‐up and outcome ascertainment were performed by reviewing the electronic health records and imaging through April 30, 2025.

Patients with sinonasal solitary fibrous tumors (SFT) were included, as they are considered borderline low‐malignant tumors, known for their tendency to recur [17, 18, 19, 20]. Exclusion criteria included tumors located at the nasal vestibule, sinonasal hematolymphoid neoplasms, and patients treated elsewhere for recurrences.

Cases were identified by reviewing the multidisciplinary tumor board meetings, during which all newly diagnosed head and neck cancer patients are discussed. Relevant patient information was subsequently extracted from electronic health records. The study conformed to the Declaration of Helsinki and was approved by the Medical Ethics Committee of the UMCU (reference no. 22‐859). Patient consent was not required under Dutch regulations, as the study did not fall under the scope of the Medical Research Involving Human Subjects Act (WMO).

### 2.2 Study Objectives and Endpoints

The primary objective was to characterize the spatial pattern of the first local recurrence after curative‐intent RT for SNM. Local recurrences were classified as in‐field, marginal, or out‐of‐field based on the dose at the recurrence center of mass (COM) after rigid registration to the pretreatment planning CT. Secondary objectives were to describe time to first local recurrence, to evaluate the contribution of perineural spread (defined as thickening or enhancement on magnetic resonance imaging [MRI] or clinical symptoms of nerve function loss) to out‐of‐field failures, and to assess the association between recurrence (any and local) and disease‐specific survival (DSS).

### 2.3 Operationalization

Patient age was determined from the date of histopathological diagnosis. Clinical variables (symptoms, medical history, smoking) were extracted from initial consultation records in the electronic health records. Tumors were staged according to the eighth edition of the Union for International Cancer Control (UICC) TNM classification for tumors of the nasal cavity and paranasal sinuses [21], with restaging from the seventh edition when applicable. Mucosal melanomas were staged per the eighth edition of the UICC TNM classification for malignant melanoma of the upper aerodigestive tract [21], and olfactory neuroblastomas via Kadish‐INSICA [22], and sarcomas per the soft tissue tumor staging system [21].

### 2.4 Diagnostic Work‐Up

Patients with a suspected or confirmed SNM are typically seen within 1 week following referral to the UMCU. Imaging generally includes computed tomography (CT) and MRI of the head and neck, chest x‐ray (or chest CT), and neck ultrasound with fine‐needle aspiration cytology of suspected lymph nodes. PET–CT was performed when the risk of distant metastases was expected to be high. Histological diagnosis was confirmed by a specialized head and neck pathologist. All patients were discussed at a multidisciplinary tumor board. Posttreatment follow‐up was scheduled periodically over 3–5 years, depending on histology.

### 2.5 Imaging and Treatment of Primary Tumor

#### 2.5.1 Surgery

In patients undergoing endoscopic surgery, the goal was complete tumor resection. In cases of uncertainty regarding resection margins, intraoperative frozen sections were obtained, and additional resection was performed at that margin.

#### 2.5.2 RT

All the patients included in this study were scanned with a mask. All patients have received multimodal therapy, being either primary (chemo)radiotherapy, induction chemotherapy followed by (chemo)radiotherapy, or surgery with postoperative (chemo)radiotherapy. Postoperative radiation started within 6 weeks after surgery.

For RT target delineation, an institutional protocol was used for all patients. This protocol was unchanged during the study period.

Gross tumor volume (GTV) was defined as a visible tumor on imaging (CT, MRI, PET/CT) and endoscopy findings. For patients treated with induction chemotherapy, the pre‐chemo volume was considered GTV. Clinical target volume (CTV) for primary cases was defined as GTV + 5 mm, cropped to anatomical borders. In case of macroscopic perineural growth, the affected nerve was included in the CTV at least until the base of the skull. For adenoid cystic and SCC (known for its perineural growth), the protocol stated that cranial nerves in proximity to the primary tumor should be included in the CTV up to the base of the skull. For postoperative cases, the CTV was defined as original tumor volume + 5 mm, cropped to anatomy. Planning target volume (PTV) was defined as CTV + 3 mm. The total dose for primary cases was typically 70 Gy in 2 Gy fractions over 7 weeks. For postoperative cases, this was 60–66 Gy in 2 Gy fractions.

RT was delivered using photons with a volumetric modulated arc technique (VMAT) with cone beam CT guidance. Patients were typically treated with 2 Gy fractions.

#### 2.5.3 Follow‐Up

At the UMCU, head and neck cancer patients who receive RT enter a dedicated MR follow‐up protocol. For this purpose, the patients' custom‐fit five‐point thermoplastic immobilization masks and individualized head supports were stored and used for all follow‐up imaging.

The standardized imaging protocol was to have the pretreatment MRI and/or PET–CT in the mask, then 3 months after the end of treatment, and yearly afterward. MR imaging is performed on a Philips Ingenia 3.0 T machine. The standard protocol is to obtain a T1‐weighted image (with and without gadolinium), an mDixon TSE T2‐weighted image, and diffusion‐weighted images (DWI).

For DWI, the SPLICE DWI sequence was used because it limits geometrical distortion so that these images can be used for delineation [23].

The RT masks are stored for at least 2 years after the patient's treatment, based on the previous research at our institution, which demonstrated that 95% of recurrences are diagnosed within 20 months after treatment [24].

#### 2.5.4 Recurrences

Recurrence was defined as radiologic and/or histologic evidence of disease relapse after completion of primary treatment. Recurrence was confirmed histologically whenever feasible; otherwise, recurrence was established by multidisciplinary consensus based on serial imaging, endoscopy, and clinical course.

All imaging was matched to the pretreatment CT, which served as the basis for the delineations of target volumes. Registrations across different imaging modalities were performed using MiM software (GE Healthcare, Beachwood, OH, USA). Since imaging is performed with the individual patients' RT masks, accurate registration is possible with only rigid transformations without rotations. In this study, we used automatic box matching by selecting the whole head in most cases when the RT mask was used for imaging or selecting the boxed volume around the recurrence to facilitate a more accurate local match when necessary.

Patients with local or locoregional recurrences were delineated by a radiation oncologist and head and neck surgeon using a standardized method by completing a delineation checklist for every patient. To reduce information bias, delineations and recurrence classification were performed using a predefined workflow and thresholds, and uncertainties were resolved by consensus. The aim was to use all the necessary information available, including physical examination, endoscopy results, and all the imaging available (recurrence and follow‐up MRI and/or PET–CT), radiologists' report with guidelines pointing to the recurrence, and pathology reports, and to delineate recurrence on all slices.

The analysis of the recurrence location is based on the COM approach, in which the COM is assumed to be the origin of the recurrence. Since all imaging was in the RT position, the recurrence imaging was rigidly matched to the pretreatment imaging and the subsequent treatment plan. The dose at the COM was determined. All COMs receiving > 95% of the prescribed dose were considered in field, those receiving 90%–95% of the dose were considered marginal, and those receiving < 90% were considered out‐of‐field.

### 2.6 Statistical Analysis

Statistical analyses were employed using IBM SPSS v31.0. Normality of data was assessed through Q–Q plots and Shapiro–Wilk tests. Data were reported as either medians with the 25th and 75th percentiles for non‐normally distributed variables or means with standard deviations for normally distributed variables. Survival rates at 3‐ and 5‐year intervals were estimated using the Kaplan–Meier estimator. Follow‐up duration for all outcomes was from diagnosis to last contact or event date, with the cutoff at April 30, 2025. For overall survival (OS), the event was death from any cause. For DSS, the event was death attributed to SNM; deaths from other causes were censored at the date of death.

Survival time was calculated from the end of RT until the event.

## 3 Results

A total of 141 SNM patients treated with curative intent were identified; 104 patients underwent RT (postoperative *n* = 93, primary *n* = 11). All patients underwent endoscopic surgery. Macroscopic complete resection (i.e., negative frozen sections) was achieved in 70 of 93 (75%) of the patients.

Of the 11 patients that underwent primary RT, in 5 patients concomitant chemotherapy was administered (2 × cisplatin 100 mg/m^2^, and 3 cisplatin 80 mg/m^2^, and etoposide 100–120 mg/m^2^). In the adjuvant setting, in five patients, concomitant chemotherapy was added as radiosensitizer (cisplatin 100 mg/m^2^ [*n* = 4] and carboplatin [*n* = 1]).

The PTV coverage (V95% > 95%) was met in 75% of the patients. In 25% of patients, there was an underdosage in favor of OAR dose; of this group, the portion of a V95% PTV coverage of 90%–94% was 65%. The lowest V95% PTV coverage was 84% because of intracranial growth and underdosage in the brain.

Median follow‐up for the total cohort was 38 months (IQR: 18–60 months).

Patient characteristics are summarized in Table 1.

**TABLE 1:** Characteristics of SNM patients treated with radiotherapy.

	Postoperative (*n* = 93)	Primary (*n* = 11)	Total (*n* = 104)
Histology, *n* (%)			
SCC	32 (34%)	5 (45%)	37 (36%)
ITAC	19 (20%)	0	19 (18%)
SNUC	9 (10%)	1 (9%)	10 (10%)
SNMM	6 (6%)	0	6 (6%)
ONB	7 (8%)	1 (9%)	8 (8%)
AdCC	4 (4%)	1 (9%)	5 (5%)
Other^a^	16 (17%)	3 (27%)	19 (18%)
cT‐stage, *n* (%)			
T1	6 (6%)	0	6 (6%)
T2	14 (15%)	1 (9%)	15 (14%)
T3	23 (25%)	0	23 (22%)
T4a	30 (32%)	7 (64%)	37 (36%)
T4b	20 (22%)	3 (27%)	23 (22%)
pT‐stage, *n* (%)			
pT1	5 (5%)	NA	
pT2	15 (16%)	NA	
pT3	20 (22%)	NA	
pT4a	28 (30%)	NA	
pT4b	25 (27%)	NA	
Neck irradiation, *n* (%)			
Yes	26 (28%)	6 (55%)	32 (31%)
No	67 (72%)	5 (45%)	72 (69%)
Prescribed dose, mean (SD)	64.2 (7.7) Gy	65.5 (8.0) Gy	
Recurrence, *n* (%)			
Yes	49 (53%)	5 (45%)	54 (52%)
No	44 (47%)	6 (55%)	50 (48%)
Recurrence location, *n* (%)			
Local	22 (45%)	2 (40%)	24 (44%)
Regional	2 (4%)	0	2 (4%)
Locoregional	1 (2%)	1 (20%)	2 (4%)
Distant	24 (49%)	2 (40%)	26 (48%)

Abbreviations: AdCC, adenoid cystic carcinoma; Gy, Gray; ITAC, intestinal‐type adenocarcinoma; NA, not applicable; ONB, olfactory neuroblastoma; SCC, squamous cell carcinoma; SD, standard deviation; SNM, sinonasal malignancy; SNMM, sinonasal mucosal melanoma; SNUC, sinonasal undifferentiated carcinoma.

^a^ Other consists of: NUT‐carcinoma, adenocarcinoma NOS (SNAC), neuroendocrine carcinoma (SNEC), HPV‐related multiphenotypic sinonasal carcinoma, teratocarcinoma sarcoma, mucoepidermoid carcinoma, biphenotypic sinonasal sarcoma.

### 3.1 Local Recurrence Details

Of the 104 patients who underwent RT, 26 patients (25%) developed local recurrence. Median time from primary diagnosis to first recurrence was 15 months (IQR: 7–29 months). Of these, 92% (24 patients) were isolated local recurrences. Two patients (type: SCC and SMARCB1‐deficient carcinoma) developed a local recurrence synchronous with regional metastases.

The portion of local recurrence in patients treated with primary RT was 3 of 11 (27%) versus 25 of 93 (27%) in patients treated postoperatively with RT (*p* = 0.845).

In univariate analyses, it was not possible to associate factors with an increased risk of local recurrence. The results of these univariate analyses are shown in Table S1. It was not possible to include histology in the analysis due to the low number of cases per histological subtype.

**FIGURE 1:**
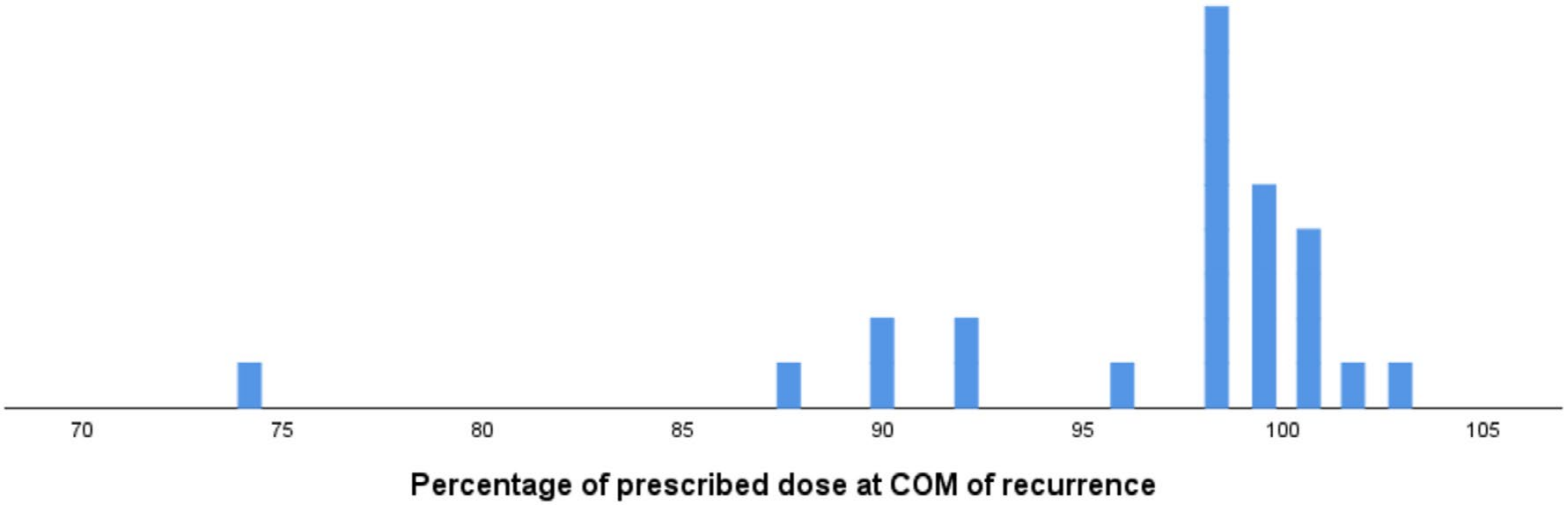
A distribution of dose at the center of mass (COM) of the recurrence. The dose is shown as a percentage of the prescribed dose. [Color figure can be viewed at wileyonlinelibrary.com]

**FIGURE 2:**
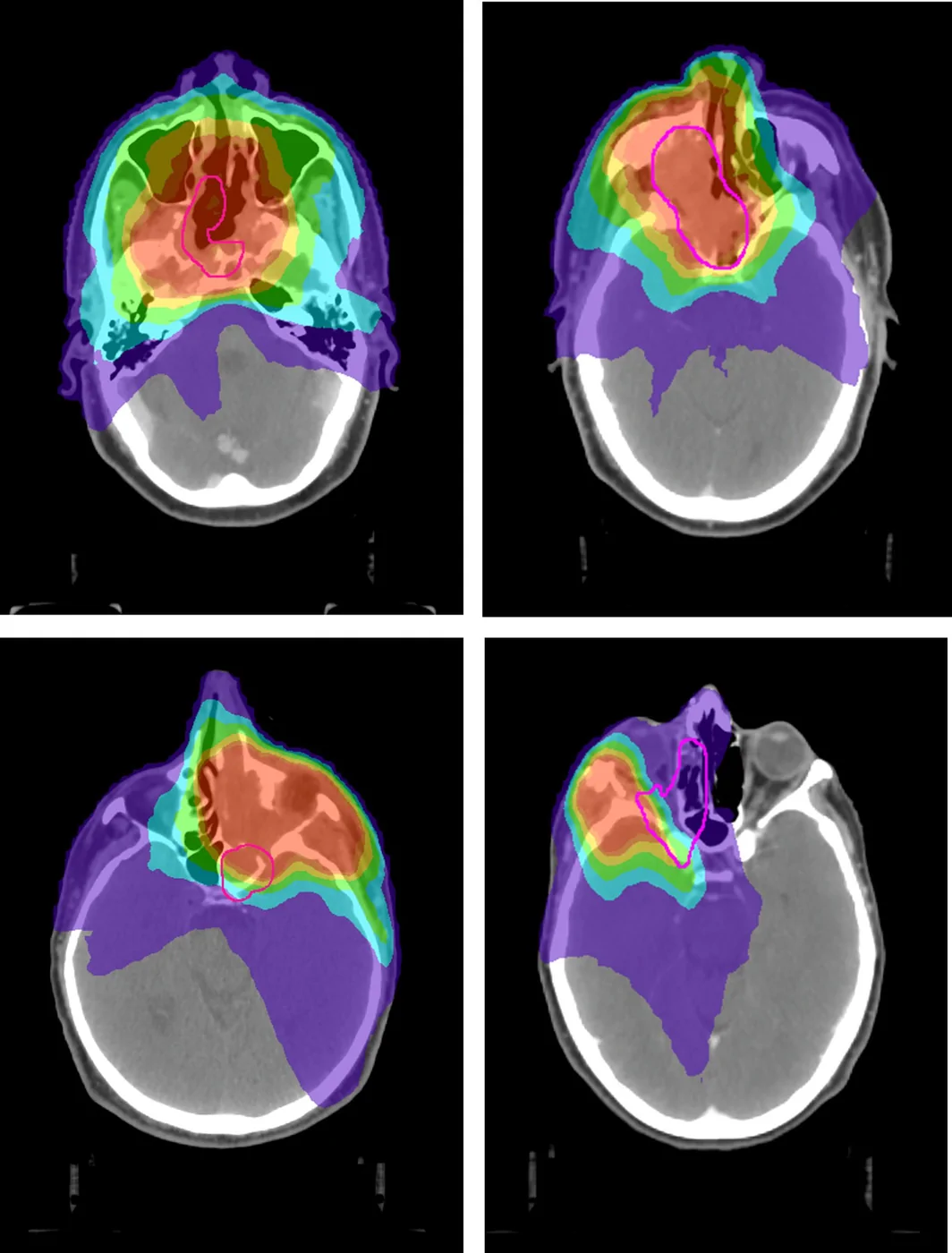
Four examples of recurrences (pink) with on the initial CT‐scan with dose overlay (red = 95% isodose). Top row 2 patients with in‐field recurrences. Bottom row 2 patients with marginal (left‐hand side) or out‐of‐field (right‐hand side) recurrences. [Color figure can be viewed at wileyonlinelibrary.com]

The median dose at the COM of the recurrence was 65.2 Gy (range: 51.9–70.4 Gy). In 21 patients (81%), the dose at the COM exceeded 95% of the prescribed dose and was considered in‐field recurrences; in 2 (8%), it was between 90% and 95% of the prescribed dose, and those were considered marginal recurrences (see Figure 1). In three patients (11%), the dose at the COM of the recurrence was less than 90% of the prescribed dose and was classified as an out‐of‐field recurrence. Illustrative examples are shown in Figure 2. Notably, two of these out‐of‐field patients had SCC with radiological perineural invasion along the optic nerve and cranial nerve V2/V3, while one had olfactory neuroblastoma with intracranial extension. There was no significant difference in this distribution between patients treated with primary RT versus postoperative RT (Fisher's exact *p* = 0.539).

Among all patients with local recurrence, 8 (31%) exhibited macroscopic radiological perineural spread involving CN V2, CN V3, and/or CN II.

Treatment of local recurrences was surgery (*n* = 9), palliative reirradiation (13 × 3 Gy) (*n* = 3), palliative systemic treatment (*n* = 9), or best supportive care (*n* = 6).

Of the patients who underwent salvage surgery, 5 (56%) developed a second recurrence. Median time to second recurrence was 37 months (IQR: 33–51 months). Histology of the patients with a second recurrence was: SCC (*n* = 2), ITAC (*n* = 1), SMARC B1 (*n* = 1), and MM (*n* = 1). All these second recurrences were isolated local recurrences.

### 3.2 Survival After Local Recurrence

OS of the entire cohort at 3 and 5 years was 68% and 45%, respectively. There was no difference between patients who underwent primary RT versus postoperative RT (*p* = 0.461).

**FIGURE 3:**
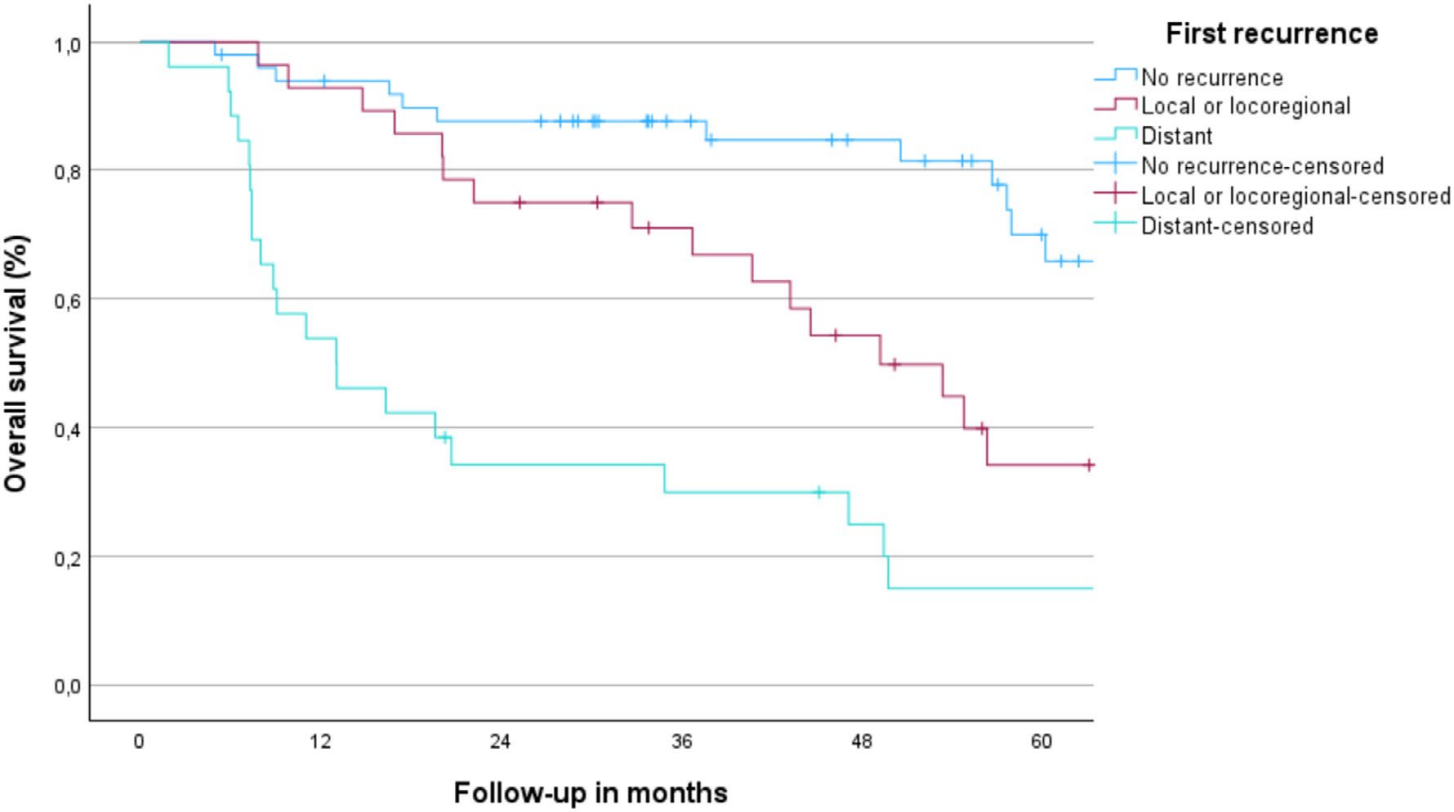
Overall survival (OS) in months for patients with and without local recurrent disease (*p* < 0.001). [Color figure can be viewed at wileyonlinelibrary.com]

Patients with local or locoregional recurrent disease had a significantly lower (*p* < 0.001) survival compared to patients without recurrence (5‐year survival 34% vs. 66%). Patients with distant metastases had the worst OS with a 5‐year survival of 15% (Figure 3).

Of the 26 patients with local recurrent disease, 15 (58%) died due to progression of disease. The 3‐ and 5‐year DSS rates of this group were 65% and 34%, respectively.

## 4 Discussion

This study demonstrates a local recurrence rate of 25% after curative RT for sinonasal cancer, albeit primary or postoperative. Local recurrent disease had a significant impact on DSS. In our series, half of all recurrences were local recurrences, and local recurrences had a detrimental impact on DSS. Crucially, regarding the current management of SNM, most of the local recurrences (over 80%) occurred within the high‐dose region. This suggests inherent radio resistance to the current total dose (60–70 Gy) and fractionation regimens (2 Gy per fraction) currently used in the treatment of SNM as a group. This finding aligns with prior studies, notably the DAHANCA study by Sharma et al. [25] and a Chinese study by Wang et al. [26]. The DAHANCA study is similar to ours, as it evaluated all patients with SNM and evaluated the pattern of recurrences in relation to the irradiated volumes and doses. The Chinese study specifically evaluated SN SCC.

DAHANCA studied 39 patients with recurrent disease after RT and found that 74% was within the original CTV. The median dose at the point of origin was 64 Gy. They also found that the criteria for target coverage (V95%) in the initial treatment plan were not met in 48% of the 184 patients. The reason they gave was sparing critical OARs, such as the optic apparatus and the brainstem. In their analysis, they found that 26% of recurrences occurred outside the CTV. In our series, only three recurrences (11%) were found outside the initial target volume, and these were all due to perineural spread or intracranial outgrowth. There were no mucosal recurrences outside the CTV.

The Chinese study evaluated 214 SCC recurrences of a total cohort of 441 (recurrence rate 48%). Of the recurrences, 65% were local recurrences, and of the local recurrences, 55% were in the field, 34% were marginal, and 11% were out of the field. However, their definitions were slightly different from ours and the DAHANCA definitions. Local recurrences in Wang et al. were categorized as in‐field, marginal, or out‐of‐field if > 95%, 20%–95%, or < 20% of the volume of failure, respectively, was encompassed by the 95% prescription isodose curve of the PTV. At the same time, DAHANCA and the present series used stricter criteria (COMs receiving > 95% of the prescribed dose were considered in the field, those receiving 90%–95% of the dose were considered marginal, and those receiving < 90% were considered out of the field).

Methods to analyze recurrence after RT can be based on comparing volumes or point‐based methods. Volume‐based quantification of the overlap of the volume of the recurrence with the initial target volume [27]. The most used method is the one used in Wang et al., as described above. The biggest advantage of this method is that it is relatively easy and reproducible. However, the volume of the recurrence can be biased by delay; if the diagnosis of recurrence is late, the volume could appear out‐of‐field or marginal, while the recurrence originated in‐field.

Point‐based methods, like the COM approach, evaluate recurrences as a concentric expansion around a radioresistant core [24]. This method is less dependent on the delay of diagnosis, but more dependent on the registration accuracy of the planning CT and imaging of the recurrence [28]. Also, the COM approach may not capture the true radioresistant core within heavily irregular shapes.

Then there is the combined approach introduced by Mohamed et al., the method we applied in our study as well, in which the point‐based analysis was combined with dose distribution evaluation [29]. This method is more complex, and since the point/dose relationship is deduced to one voxel, the registration accuracy becomes more important than in volumetric methods. To limit this error in the present study, patients underwent all imaging in an RT immobilization mask.

Current ESTRO and ASTRO head and neck guidelines only cover mucosal HNSCC or salivary gland cancers. Subsequently, it leads to practice variation and interobserver variability in target delineation for the SNM subgroup. The French REFCOR group published a consensus statement on sinonasal cancer, also covering recommendations regarding RT [30]. Internationally, there is consensus on the need for close communication between surgeons and radiation oncologists to adequately define the CTV after surgery [31]. Also, the French group from REFCOR performed a Delphi consensus study on a delineation atlas for sinonasal substructures after endoscopic skull base surgery [32]. These efforts notwithstanding, there is an urgent need for histology‐driven guidelines for RT in SNM.

Another critical aspect is biological radioresistance to the current RT schemes, which vary among histological subtypes. Thinking of SNM as a group (and treating them as such) has been the treatment protocol to date. However, the current sinonasal histology‐driven surgery and medical oncology treatment movement could open up discussion whether this should also be applied for RT [11]. Other oncology fields, outside of sinonasal cancer, have already adopted a histology‐driven approach. It has been shown that cutaneous melanoma is radioresistant when treated with a fractionation scheme of 2 Gy per fraction, but (extreme) hypofractionated RT could result in favorable outcomes [33]. This could be a rationale for future SNMM research. The same holds true for gastrointestinal adenocarcinomas. It is known that they are relatively resistant to 2 Gy fractions and respond better to hypofractionated schemes, which has likewise been shown in pancreatic cancer or colon carcinoma [34, 35]. This raises the question of whether ITAC or other SN adenocarcinomas could respond better to a more hypofractionated approach. This could also be used as a rationale for future research.

The resistance of the various tumor types to RT can also be tested using organoids derived from tumor tissue of the SNM. This can be done for groups of tumor types, but also individually. It can be anticipated that if the tumor appears to be radioresistant, RT can be omitted or intensified [36].

Given the rarity of SNM, randomized trials stratified by histology are impractical. Instead, pragmatic approaches—such as adapting RT schemes from other tumor sites—may offer a feasible pathway to improve outcomes. To do so, developing technical tools to deliver alternative fractionation schedules, independent of histological type, is essential.

Limitations of our study include its retrospective design, monocenter design, and the lack of detailed information on prognostication. Inherently, the numbers are low, limiting statistical power to define risk groups. However, the purpose of our analysis was to evaluate the location of recurrent disease in relation to the RT dose.

## 5 Conclusions

Our findings indicate that local recurrences of SNM predominantly occur within high‐dose regions, highlighting the challenge of radioresistance with the currently applied dose/fractionation schemes. There is an urgent need for standardized delineation guidelines, biologically tailored RT schemes, and innovative technical solutions to improve local control. These findings are hypothesis‐generating and support the development of histology‐specific, biologically driven RT strategies, which warrant prospective validation.

## Funding

The authors have nothing to report.

## Supporting information


**Table S1:** Univariable Cox regression analyses of risk local recurrence.

## Data Availability

The data that support the findings of this study are available on request from the corresponding author. The data are not publicly available due to privacy or ethical restrictions.
