# Supplementary material for: Predominance of In‐Field Recurrence After Radiotherapy for Sinonasal Cancer: A Single‐Center Retrospective Study
Source: Head Neck. 2026 Apr 23;48(10):2704–12. doi: 10.1002/hed.70287 (PMC13595468; doi:10.1002/hed.70287)
Supplement: Supplementary file 1 [file HED-48-2704-s001.docx]

Supplementary table 1 – Univariable Cox regression analyses of risk local recurrence

|  |  | HR local recurrence (95% CI) | p-value |
| --- | --- | --- | --- |
| T-stage | T1-2 | Ref |  |
|  | T3 | 1.69 (0.57-5.05) | 0.346 |
|  | T4a | 0.57 (0.15-2.12) | 0.400 |
|  | T4b | 1.68 (0.55-5.14) | 0.365 |
|  |  |  |  |
| Surgical margins | Complete | Ref |  |
|  | Macroscopic residue | 0.94 (0.37-2.37) | 0.902 |
|  |  |  |  |
| Treatment | Primary RT | Ref |  |
|  | Surg + RT | 1.21 (0.3-4.01) | 0.755 |
|  |  |  |  |
| Dose coverage | Adequate | Ref |  |
|  | Underdosed | 1.72 (0.73-4.03) | 0.214 |
|  |  |  |  |
